# Spatial Syndromic Surveillance and COVID-19 in the U.S.: Local Cluster Mapping for Pandemic Preparedness

**DOI:** 10.3390/ijerph19158931

**Published:** 2022-07-22

**Authors:** Andrew J. Curtis, Jayakrishnan Ajayakumar, Jacqueline Curtis, Sam Brown

**Affiliations:** 1 Department of Population and Quantitative Health Sciences, School of Medicine, Case Western Reserve University, Cleveland, OH 44106, USA; ajc321@case.edu (A.J.C.); jxa421@case.edu (J.A.); 2 University Hospitals, Cleveland, OH 44106, USA; sam.brown@uhhospitals.org

**Keywords:** COVID-19, GIS, spatial epidemiology, vaccine

## Abstract

Maps have become the de facto primary mode of visualizing the COVID-19 pandemic, from identifying local disease and vaccination patterns to understanding global trends. In addition to their widespread utilization for public communication, there have been a variety of advances in spatial methods created for localized operational needs. While broader dissemination of this more granular work is not commonplace due to the protections under Health Insurance Portability and Accountability Act (HIPAA), its role has been foundational to pandemic response for health systems, hospitals, and government agencies. In contrast to the retrospective views provided by the aggregated geographies found in the public domain, or those often utilized for academic research, operational response requires near real-time mapping based on continuously flowing address level data. This paper describes the opportunities and challenges presented in emergent disease mapping using dynamic patient data in the response to COVID-19 for northeast Ohio for the period 2020 to 2022. More specifically it shows how a new clustering tool developed by geographers in the initial phases of the pandemic to handle operational mapping continues to evolve with shifting pandemic needs, including new variant surges, vaccine targeting, and most recently, testing data shortfalls. This paper also demonstrates how the geographic approach applied provides the framework needed for future pandemic preparedness.

## 1. Introduction

Teasing out a geographic understanding of COVID-19 (C19) pattern and diffusion has paralleled the course of the pandemic [1,2,3,4,5]. In keeping with the aims of this special issue, there has also been considerable operational need for the analysis and visualization of granular C19 data to solve near-real time emergent threats to local populations [6]. In this paper we explore this topic in terms of the spatial data analytics involved in the day-to-day health operations of a multi hospital collaboration. In this setting, working with dynamic granular health data and trying to make sense of emerging patterns in near-real time so that response strategies could be mobilized posed considerable technical, cartographic and spatial analytical challenges. Shifting needs across the pandemic included where to target health team interventions, where to guide on-the-ground testing or vaccine resources, or in 2022, how to extract diffusion signals in a spatially biased testing landscape. The typical spatial analytical needs of an “operations model”, such as is typically found in an incident command structure implemented for these types of massive externalities [7,8,9], whether in a hospital, health department or a state level response, are different to more prospective “research”. While geospatial research into C19 can provide valuable insights [10,11,12] gained through a more reflective, and theoretically deeper lens, these studies do not face the continually shifting data, epidemiological, economic, and even political environments in which the operations world sits.

Yet it is vital that we understand these types of geospatial applications so they can be critiqued, triage advances disseminated, and potential data quality issues understood in case they propagate into the research world. Indeed, understanding how the spatial response to C19 had to change during the pandemic helps to contextualize temporal and spatial biases in subsequent data used for analysis. In this paper we provide a window into that spatial operational perspective, including the development of new analytical and visualization advances that helped make sense of the within-pandemic continuous disease data flow. In so doing we will show how GIScientists supported the C19 response for one state and how their work has created an ongoing model for future pandemic preparedness. We will also outline the data challenges faced and how these might have implications for subsequent geographic pandemic research and will describe the analytical and technical advances that continue to be made to solve those data puzzles. While there are a number of excellent articles detailing geospatial methods and their applications in C19, they have primarily been written within the domain of science. The work presented here is different in that it is the result of a scientific-operational collaboration. As such, we present proof of principle and the higher level thinking that led to its development so that it can be replicated across hospitals and health systems.

At a recent National Science Foundation (NSF) sponsored workshop on Predictive Intelligence for Pandemic Prevention (NSF-PIPP 2021), the authors of this paper presented a relatively common stance found in disaster response, that in order to prepare for the “next one”, lessons learned had to be recorded, disseminated and transformed into proactive action steps [13,14,15,16]. During the end of 2021, the United States had faced a massive surge of C19. Ohio in particular had one of the highest infection rates as the Delta and Omicron surges blended together. Once this surge had waned in the beginning of 2022, the region settled into a new-normal of looking for new outbreaks (and new variants), while society as a whole started to move away from the behavioral preventative measures. The tools needed to lessen C19’s severe health impact had improved, with the tool chest now including vaccines, prophylactics, and an almost society wide level of exposure from the Omicron surge. However, while the stress on hospital systems continued to lessen, there was still an understanding that ongoing syndromic surveillance would be required to catch what would come next. Adding challenge to this task was that many of the data inputs that had proved vital in identifying emerging patterns in 2020 and 2021, including widespread C19 testing, signals from severe hospital admissions, and Emergency Medical Services (EMS) run data were becoming less reliable and more geographically biased. While alternative data sources, especially wastewater, provided some hope to fill the gap, logistical challenges involving its sampling, and the delay in getting and then analyzing samples meant that in early 2022 it was still a less than optimal support for operational decision making [17,18,19,20,21].

Adding further complexity to teasing patterns from any available C19 data are a continuously shifting set of societal responses including variable lockdowns, mask mandates, social-distance guidelines and most importantly a reluctance along socio-cultural and political lines for testing. The evolving vaccine landscape adds further geographic challenges [22], especially as the protection it offers is not binary; the percentage of people who have received at least one shot, those who are fully vaccinated (and does this just mean two shots?), and the percentage having received first and second boosters all result in different levels of risk. These data are also nuanced by the date of the shot, especially when considering different age cohorts. The overall impact is a continually changing risk map and being able to track this level of spatial complexity in near real time requires analytical creativity. Indeed, as a result of all these factors, understanding the localized disease risk including the amount of disease, and the vulnerability of the surrounding population, is as challenging in 2022 as it has ever been during the pandemic.

A spatial approach has always been integral to the C19 response in northeast Ohio. As with most municipalities across the United States, the specifics of that spatial response, from employing basic mapping to using spatial analysis, was largely dependent on the skillsets and prior experience of the local data analytics team. Federal, state, academic, and even private data dashboards did provide valuable mapped insights as to the overall problem, including how the region fluctuated with respect to its neighbors [23,24,25]. For example, the Ohio Department of Health (ODH) COVID-19 Dashboard (https://coronavirus.ohio.gov/wps/portal/gov/covid-19/dashboards/key-metrics/cases-by-zipcode, accessed on 21 July 2022) provided a publicly available download of daily zip code level case data for the preceding 14 day period. Eventually this daily download reverted to a once-a-week update, at least in part because of the perceived reduction in data quality through the use of non-reportable home tests. Even so, mapping these data revealed the ebb and flow of positive case data in the region. Diffusion patterns could be assessed, again depending on the expertise of the available data scientists. For example, some surges in the summer or early fall, spread originated in the south of the state and moved northward, while at other times it diffused from Michigan to the northwest, and/or New York and then Pennsylvania from the east. In mid-2022 a more hierarchical diffusion occurred around the main population centers, especially Columbus and Cleveland.

**Figure 1:**
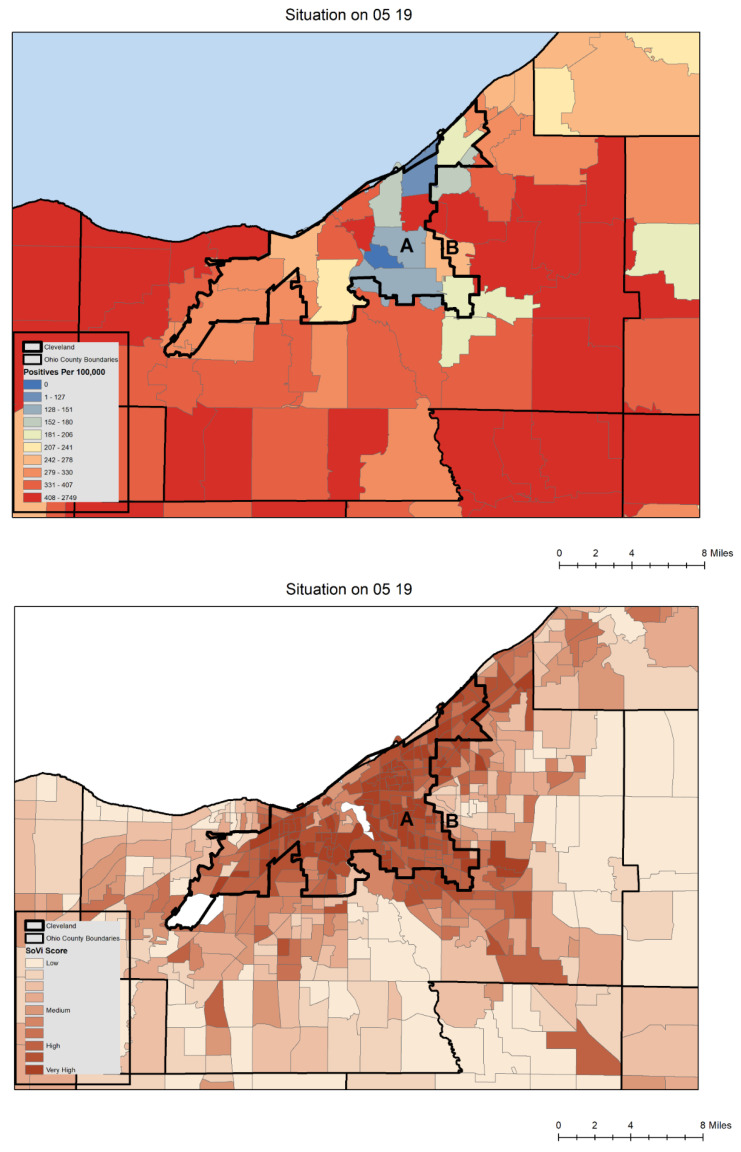
Zip Code mapping of positive case data per 100,000 population for the zip codes of Cuyahoga County and mapped social vulnerability for the same map extent. The CDC’s social vulnerability measure (https://svi.cdc.gov/map.html, accessed on 21 July 2022) was often used as a backcloth for the production cartography shared with health departments and community groups to help contextualize output. In this figure the blue zip codes suggest an area of little to no disease, where in reality it is a highly socially vulnerable section known locally as the “Cleveland Crescent” which coincided with historically red lined areas (areas that had been designated as undesirable for real estate investment due to the presence of minority and low-income populations in the 1930s). The cold spot is indicative of low levels of COVID-19 testing. Source: GIS Health & Hazards Lab, School of Medicine, Case Western Reserve University.

These dashboard data could also reveal potential problems and biases in what was being reported, especially as testing became less widespread in later 2021 and 2022. Most notably, areas with high social vulnerability scores in the large urban centers would map as having little disease which is counter to what is expected [26,27,28]. The erroneous map interpretation of these cold spots would have been that the more susceptible areas were being spared, whereas the reality was the map pattern was showing where there were testing deficiencies (Figure 1). During the surges of 2021, approximately two weeks later, the map would infill as more serious cases (and then their families) would be tested. But this delay in the signal had consequences in communicating local risk. Consider Figure 1 from May 2022 which shows positives normalized by 100,000 residents for the previous 14 days. Most of Cuyahoga County are represented by various shades of red, contrasting with the cold area showing up as blue and labelled “A” which also corresponds to the most socially vulnerable areas of Cleveland shown on the lower map. Again, an erroneous interpretation of this map might be that the more socially vulnerable area (the darker browns on the lower map) was not experiencing disease because the area had suffered so greatly in previous surges. A different erroneous interpretation would be that the vaccine strategies in this area had been extremely successful. Both explanations have previously been offered by some in public health to explain this type of map. The reality is (as is proven by mapping out the individual level negative and positive test data available through the soon to be described hospital collaboration) that people living in this area are not getting tested for a variety of reasons. Not only are these data problems challenging for an accurate reading of the situation, which is vital for the local hospital systems, but also for any subsequent spatial work looking at the impact of C19 on socially vulnerable populations [29]. Yet these data are the most “official” recording of C19 in Ohio for this date. Even given these limitations, releasing data in such a publicly transparent manner should be a template for any future pandemic response, and arguably even for other health challenges such as opioid overdoses.

While the previously mentioned release of data was invaluable, it should be remembered, however, that zip code level aggregations are not appropriate for any serious analytical insight [29]. To illustrate this point, consider the zip code identified as “B” which straddles the City of Cleveland and a far more affluent neighbor, Shaker Heights (Figure 1). The color is orange, which probably neither reflects the low testing on the Cleveland side, or the higher testing (and positives) on the Shaker Heights side. Until March 2022, vaccine results had only been distributed to community organizations involved in testing at the zip code aggregation, and as this map shows, certain areas failed to show an accurate rate based on this zip code level of smoothing. To provide meaningful spatial support for operations, far more granular insights were needed.

## 2. A Combined Hospital System Response

As for much of the U.S., the people of Ohio are cared for by a patchwork of competing private health systems, with specific populations under a public single payer system, such as Medicare, Medicaid, and the Veterans Administration. As a result, because individual level patient data are distributed across different systems, any single hospital will only have a piece of any rapidly developing health condition, such as a highly contagious virus. Although these data are officially reported to a state health department, which in turn makes them accessible to local health departments, the cadence of this data flow is not aligned with emergency service on-the-ground need. More specifically there is between a 3 and 7 day (sometimes longer) delay in the positive test result routing from a lab to ODH, and then in dissemination either out to the public or to local health departments. That lag effect also has a geography, with certain regions, especially rural areas, having a relatively slower process [30]. This is no-one’s fault, but it does mean there is geographic uncertainty in the accuracy of C19 reporting on the day of reporting, with the level of uncertainty decreasing for each subsequent day. It should also be remembered that any federal or national level dashboard is limited by this same lag effect because they are at the next level of the data hierarchy, and any problems experienced at the local to state level will also propagate upwards. Therefore, while ODH conceptually has the most complete data picture of Ohio, it is neither granular enough nor timely enough for the regional/local professional health response. A delay of even a few days is a missed opportunity to map and target interventions that could save lives or understand where an emerging pattern might stress a local clinic, or where to scale back, or up, C19 testing or vaccine delivery. From the hospital system perspective there was a need to be able to “read” developing positive case counts in such a way that the effect on hospitalizations, ventilator and ICU use would not cripple medical resources. At the same time, these same approaches could expediently identify specific buildings, especially care homes, in order to trigger intercept teams and reduce the risk of spread [31]. While that desire was common to any hospital system, the means of implementation probably varied dramatically.

The most useful data input connected to such an operational need is the home address of a person who has tested for C19, and then filtering these data based on the result: positive or negative. Such data enables granular mapping of the number of tests, the number of positive tests, and positivity rate for any location. While in an ideal world everyone would be tested to maximize the predictive capacity of the test signal, the reality was the availability of tests fluctuated. When tests were scarce, some people who were known to be positive by symptom description alone, were not tested, while other populations (often living in the same areas) were over sampled because of their professions. This data “signal” would get worse in 2021 when certain cohorts no longer wanted to be tested, meaning that large swaths of major urban areas were hotspot free (as shown in Figure 1), at least until more symptomatic severe cases emerged. The early signal was now being lost.

A hospital system is likely to perform a sizeable amount of testing, and not just for their primary patients. The results of these tests populate their internal databases in near real time. Furthermore, for those tests linked to their patient population, additional insights such as detailed patient histories, including C19 related comorbidities, and the severity of the current illness, allow for a contextualized understanding of emerging disease risk. Near real time analysis of these data are vital in being able to manage stretched hospital facilities and where to target response teams as situations emerge [31]. By mapping these data, early insights may be achieved into the geography of disease transmission, the associated individual and community level risk factors, and even where new variants are emerging. However, not all hospitals have similarly trained spatial data analysts who can utilize a standard geographic information system (GIS) and its varied toolboxes [32,33]. Some may visualize spatial output from their electronic health records using statistical packages such as “R”, or alternatively there is a reliance on existing dashboard functionality to make maps. However, to fully leverage the spatial insights from these data for near-real time operational support probably requires additional programming and automation beyond the usual database manipulations and internal dashboard use. The specifics of how this happened for any hospital, and indeed how future pandemic responses would be organized probably varies considerably. *From our experience*, northeast Ohio regional health systems and the local health departments, were left to develop whatever strategy worked best for them given their available resources (e.g., data, technology, expertise) to prioritize actions, save lives, and reallocate resources.

We decided that the approach had to be flexible enough to morph with changing situations. It needed to be scalable and with automation to prevent analyst burnout. Ideally, it had to include a specific spatial syndromic surveillance approach that could reveal changes in the disease map and not just temporal snapshots. To meet these goals in northeast Ohio for University Hospitals (UH), a spatial database was built that linked the continuously flowing hospital test data with those other important internal and external contexts. Wherever possible, data manipulation, including geocoding, spatial querying, dataset merging, analysis and cluster output were automated. While there would still be daily tasks in terms of overseeing geocoding quality, writing new queries, and managing data flow problems, this automation allowed for results to be immediately viewed on hospital dashboards, and for members of the team to focus more on investigating the context of the outputs, while also producing higher quality production cartography. The spatial response team comprised within hospital data analysts who understood the various internal databases and how they connected, a spatial data analyst who performed all programing and built the spatial database and who could write the code for mapping and querying, and a geoscientist who could correctly interpret the mapped patterns and identify potential holes and spatial biases in the incoming data pattern.

More specifically, C19 test results were extracted from the hospital system’s data warehouses, geocoded, and pushed to the spatial database using periodic batch jobs [34]. In so doing a variety of different outputs including maps, grids, and tables were generated in a continuous fashion. As much of these queries were greatly dependent on positional accuracy, a three-step geocoding validation process was implemented. Being aware that while the UH data alone provided a high quality sample, collaborating with another system would dramatically improve everyone’s regional insights. As a result, UH and the Cleveland Clinic (CC) combined resources including expertise, operational reporting, and most importantly, their data. This was a unique experience in that two hospital systems in the same service area understand that the public good through collaboration outweighed any perceived “competition”. While CC had previously been mapping their own data using dashboards, these collaborative discussions settled on the benefit of an expansion of the spatial architecture established at UH within their analytic infrastructure to enable clustering of their patients’ data. After implementation and validation, CC helped advance aspects of C19 clustering pertinent for their organization especially with regards to the visualization of their output through dashboards suitable for hospital leadership. Of even greater benefit was CCs dissemination of this new spatial approach to the next level of the Ohio hierarchy (the Ohio Hospital Association), which would eventually lead to the team being included as part of a statewide surveillance system.

Even so, there were geographic obstacles to the collaboration that needed to be overcome. In addition to sharing technical insights, and comparing cluster outputs in weekly meetings, data had to be shared at a suitable geography to create combined clusters while still maintaining patient confidentiality in reporting. The solution became another geospatial advance, the use of the nine-digit postal code level (ZIP+4), an aggregation that has not widely been used or described in research due to the technical challenges it poses for mapping and spatial analysis. To this end, code was written to merge the incoming point and line data, with all technical updates and advances being immediately shared between all collaborators. This level of granularity, though sometimes imprecise in more rural areas, could easily be tied to a single street segment or congregate building in higher density neighborhoods. The ZIP+4 geographic unit, though a purely postal delivery segment, preserved the geographic fuzziness needed by both hospital systems to maintain a degree of data exclusivity while still being granular enough to generate clusters.

Different emerging disease detection analyses were programmed to run as a daily batch job. For example, emerging clusters of cases were fed to the spatial database for further spatial and longitudinal analysis. The spatial database also enabled the automatic merging of different spatial layers/sources with these emerging clusters, including social vulnerability measures (such as seen in Figure 1), different social determinants of health such as race and ethnicity, and congregate housing footprints. This data engineering and processing framework (i.e., source data ingestion, database architecture, geocoding, cluster formation and visualization) was replicated at CC. Weekly meetings between the collaboration team focused on the cluster output maps to maintain an ongoing validation of the analytical process, and importantly, to identify any potential data flow errors, such as if one county suddenly changed for no apparent reason in terms of its emergent C19 activity.

Spatial syndromic surveillance now ran automatically on inflowing data from the two hospital systems. The analysts would perform deeper dives into the clusters, sometimes employing more sophisticated GIS cartography to communicate situational awareness to hospitals, health departments and various other municipal leaders, often detailing the developing spatial structure of the clusters and not just their location. Internally, the GIScience team utilized ArcGIS products for mapping GeoMEDD output to better inform decision makers. Output maps were evaluated for the first indications of an emergent pattern, guided in interpretation by the experience of seeing how C19 had diffused through the region in the preceding years. While these data did not represent all positive test results in the region, the belief was that there was enough shared insight to act as a syndromic surveillance data layer. This approach became even more valuable as testing began to wane, and the state scaled back dashboard reporting because of the perceived holes generated by home testing.

The spatial database also facilitated the analysis of the potential impact on critical infrastructure. The daily geocoded positive cases could be automatically counted at different distances around any building. The first use of this automatic buffering occurred as a surveillance tool for all care homes. Circular buffers of 200 m, 500 m, 1000 m, and 2500 m around the center point of the complex would capture within, emerging proximate and neighborhood risks. The center point was decided upon by taking the different addresses listed, consulting aerial imagery, and manually locating the most appropriate place for the centroid. While this initial placement was laborious, it only had to be completed once, and then served (and continues to serve) as the same address throughout the pandemic. Positive cases were summed for the previous 14 days and presented as a table. Typically, these tables would be viewed every day, and any facility with a new case would lead to hospital leadership being notified and the mobilization of the intercept team to stop the spread (See https://www.facebook.com/UniversityHospitals/videos/covid-19-uh-intercept-program/771844150235613/, accessed on 21 July 2022). While this information was also made available through an automatically refreshed dashboard, it was found, as with many other automatic outputs, that the analytics teams still needed to prioritize the findings in terms of what they saw through their experience as being the highest concern.

Variations of this same automatic buffering were used throughout the pandemic, with other examples being buffers around schools to judge the risk of reopening viewed through the lens of community spread, around hospitals to predict any upcoming surge stresses, and around public housing to target vaccines. Even new variant clusters, once identified, would be monitored using this method to see how much subsequent spread was occurring and whether that differed to the rest of the area. Future research could return to these data tables to see how the geography of C19’s basic reproduction number changed over the course of the pandemic, not just as an overall measure of spread, but to show which urban sections, or rural towns, were important as diffusion conduits.

While multiple analyses were combined for syndromic surveillance, a new form of granular clustering was created for the sole purpose of identifying emergent C19 threats called Geographic Monitoring for Early Disease Detection (GeoMEDD). The initial conceptualization for this approach to spatial syndromic surveillance has been described for 2020 [34]. However, as the spatial complexity of the C19 landscape changed, with different tasks being required by the hospitals, and with data quality varying over time, so the model evolved through 2021 and into 2022. These clusters made it possible to look for micro geographies of concern with findings being highlighted at weekly meetings with local health departments to either validate ongoing intervention strategies or initiate new intercepts. In return the local health departments would suggest analytical modifications such as identifying school-age clusters which, in the spring of 2021, would lead to the identification of new variant hotspots.

**Figure 2:**
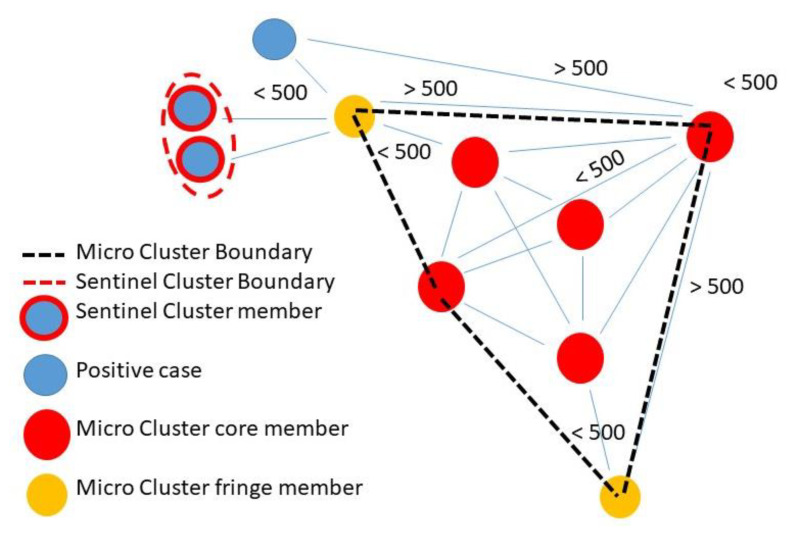
An example of *Micro* cluster formation using GeoMEDD. Five core members to the *Micro* cluster (red dots) are all within 500 m of each other. Two fringe members (orange dots) are within 500 m of one cluster member, but not all other core members. A case within 500 m of a fringe member but outside of 500 m to a core member does not grow the cluster. A *Sentinel* cluster (at least two members within 100 m of each other) is also shown. The boundary of the cluster is created by joining the centroids of all cluster members (black dotted line), or creating a 100 m ellipsoid (red dotted line) in the case of *Sentinel* clusters.

At its core, GeoMEDD is an additive clustering tool designed to identify spatial patterns and provide a hierarchical understanding of the spatial structure of clusters (Figure 2). It is a data analytics inspired tool that works with continuously inflowing data. These clustered data can be used to tease out spatial and temporal diffusion patterns in the form of important buildings, routeways and neighborhood subsections. With the addition of descriptive text, it can also provide a contextual insight suitable to support epidemiological efforts including contact tracing. Data input requires a geolocated point, which typically for the C19 response is a positive test, though patterns of patients who are hospitalized, those in the Intensive Care Unit (ICU), or even mortalities have also been used. In this way, identifying patterns of disease emergence and severity, and near-real time operational diffusion monitoring, are possible in ways not readily available from typical GIS supported clustering.

The 2021 and 2022 version of GeoMEDD still required setting a minimum number of positive cases, a maximum bandwidth to specify geographic proximity (the size of the connecting distance between positives), and a look-back time interval (Figure 2).

What changed was the justification behind those selections. Initially, and as reported for spatial syndromic surveillance in 2020, three basic cluster types included 10 positive cases within 1000 m, capturing broader *Neighborhood* trends, 5 within 500 m, for sub-neighborhood *Micro* patterns, and 2 within 100 m, often being in a single building which would give the first glimpse of emergence being a *Sentinel* indicator. In 2020 all of these were calculated every day for the previous 21-day period. However, as the pandemic changed geographically, and with new surges adding far higher volumes, new cluster types were developed. For example, in more rural areas and to account for their more linear communities often living along a single route, *Sentinel* clusters were expanded to 2 positives within 500 m. During the Delta/Omicron surge in November 2021, super *Neighborhood* clusters were utilized including 20 positives within 1000 m, and for hyper intense buildings, 10 positives in 100 m produced super *Sentinels*, which usually indicated a congregate building. These super clusters were partly due to the higher volume surges in 2021, but also the increased surveillance (and therefore greater inflowing data) when the team were tasked to provide spatial syndromic surveillance for all of Ohio using a more complete positive dataset sourced from the Ohio Department of Public Health, though with those previously described lag effects. An additional modification was the change in the look back period. While clustering in 2020 could be used to describe both the spatial pattern of the outbreak, and to extract out emergence from those trends, during 2021 attention shifted almost entirely to syndromic surveillance. Knowing where the first disease rises were occurring both helped target intervention, and also provided a 7 to 10 day forewarning of where hospital systems would become stressed, either in terms of exhausting building and equipment capacity, or potentially losing staff. To do this the look back period changed to 7 and 3 days. By combining the 3 and 7-day super, *Neighborhood*, *Micro* and *Sentinel* clusters, not only could an area of emerging positive cases be located, but the actual “drivers” (meaning disease foci within the cluster such as specific congregate buildings) were identified. For the December 2021 Omicron surge an even higher-level cluster (30 positives within 1000 m) and a shorter look back period (1 day) was added to make sense of the extraordinarily high case numbers.

**Figure 3:**
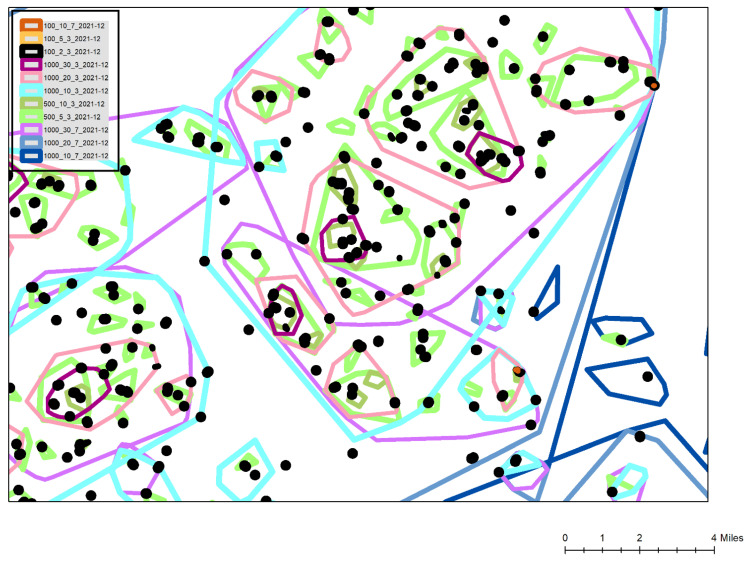
A Spatial Syndromic Surveillance cluster output from the Omicron surge using GeoMEDD.

Figure 3 is a typical cluster map created during the Omicron surge. This map is a simplified view for this publication with all associated geographic underlays, such as streets, congregate building footprints and political boundaries removed to preserve confidentiality. Also, multiple cluster types and additional attribute labels are omitted for the sake of clarity. The legend displays the included cluster types, for example 100_10_7 is a super *Sentinel* with at least 10 positives occurring within 100 m of each other in the preceding 7 days. The most commonly used *Sentinel* cluster type, the black shapes of the 100_2_3 layer show buildings or street segments that had emerged over the previous 3 days. The combination of the 100_10_7 and 100_2_3 clusters (two can be seen labelled as A on the map) show buildings where there had been a high number of cases over the last 10 days, and with at least some of those occurring in the last 3 days suggesting the diffusion was ongoing. To validate this last interpretation, these clusters were often labelled by their total cases, or by a vulnerability attribute such as the number being elderly. In this way these hyper-localized disease locations could be monitored to see if the clusters continued to grow, and even if they were spreading to neighboring buildings of which there were usually several within the same or neighboring complexes. These locations, especially when verified using aerial imagery, such as Google Earth, also provided to be useful in identifying elderly focused but privately owned congregate buildings that did not appear on the normal hospital care home lists. During later 2021 and early 2022 the identification of these buildings would also support mobile vaccine response team targeting.

Different types of *Neighborhood*, *Micro* and *Sentinel* clusters are displayed for 3 and 7 day look back periods. Under normal conditions, focus is on the light blue 3 day *Neighborhood* clusters (at least 10 positives within 1000 m during the last 3 days). On this map normally the total number of cases within the cluster is labelled along with the positivity of each cluster. Other attributes that could be labeled include the percentage of the positives being admitted, the age structure, and even the number of positives with prior C19 related health vulnerabilities. In this way each cluster not only presents a geographic “risk”, but this is contextualized by the likelihood of the cluster placing stress on the health system. During extreme surges, the super *Neighborhood* (20 within 1000 m shown as light pink) and extreme *Neighborhood* (30 within 1000 m shown as dark purple) were also utilized. Both sets of clusters would be similarly labelled with the total number of positives within the last 3 days, and the associated positivity for the cluster. In this map the super neighborhood clusters had total case numbers of 192, 189 and 78, with positivity percentages of 56, 61 and 57, respectively. General institutional guidance within the hospital system is that a positivity rate of 20% or above is severe and cause for concern. By reading the hierarchical stacking of these cluster types, even more geographic precision is possible, showing where positivity may indicate a higher level of symptomatic disease, and a potential stress on the local hospital system, especially when they occur within areas of high social vulnerability with historically low testing. This has become even more vital during 2022 when home testing, and a general apathy to testing in the typically more socially vulnerable areas requires an even more nuanced reading of the clusters. Certain areas, for example where employees are required to be tested through their employment, provide consistent areas of disease surveillance. Watching the positivity in these clusters, and then the positivity in any lagged cluster development in the socially vulnerable areas, provides an understanding of whether the disease is spreading, where problem locations are, which resources are likely to become stressed (for example by considering where hospital staff live), and just as importantly, when and where things start to improve. While more traditional spatial analytical methods provide different insights (for example a hotspot map by census tract aggregations), none provide the spatial and temporal flexibility shown here and which are vital to health system and public health operations While future work can more fully compare how different spatial analytical methods reveal different patterns in the same data, the flexibility and usability of GeoMEDD has been found to be unmatched from an operational perspective given continuously inflowing data, a variety of different “needs”, and available skill sets.

## 3. Discussion

While the pandemic is by definition global, from a public health, hospital resource, and community perspective, it is comprised of *local* outbreaks requiring *local* understanding and response [35]. Therefore, geographic data science methods need to be focused at this more granular scale and cadence of intervention to see exactly where, how and when surge happens, and then how best to intervene or bolster surrounding medical resources. The spatial syndromic surveillance approach and cluster method described in this paper was originally developed in 2020 to understand the mapped pattern of disease and identify the localized spatial structure and the drivers that can be targeted and addressed in near real-time. As the local version of the pandemic changed in northeast Ohio, especially during the later parts of 2021, so did the analytics also evolve to understand a more socially and geographically complex situation, including an uneven vaccine landscape. There was the additional need for a *near real time* and granular spatial support for mobile vaccine pods, often working through community organizations or Federally Qualified Health Centers (FQHCs) [36]. If done well, an emerging cluster could be limited in terms of spread if vaccine were targeted strategically around. Even at its simplest level, this now meant the combined spatial data required to create a risk map included percentages of who had received a first vaccine dose, those who had completed two doses, and boosters (e.g., [37]), new positive cases, all tests taken (for positivity calculations), pre-existing health and social vulnerabilities, and severity of illness. To react to this spatial complexity, there were continual innovations from a spatially informed data-science approach. Hospitals gained additional data insights from the text mining of electronic health records for symptoms, and the frequency, and details of emergency service runs. Even the placement of wastewater testing locations in the known data holes (location A in Figure 1) were added into an evolving system of spatial syndromic surveillance. Other advances included a suite of new aggregate syndromic surveillance tools, including a daily generated “heat map” of new cases based on “driver” regions identified through two years of analyzing cluster outputs, a legacy map showing where clusters had intersected each cell of a grid over the last 40 days, and a census tract map of positivity. Just as at the beginning of the pandemic, the geographic monitoring of all things C19 in northeastern Ohio continues.

Indeed, arguably, there has never been another period where geography, geospatial methods, maps, and a *spatial health science* have advanced as quickly in terms of operational utility.

While in mid 2022 severe hospitalizations and deaths are of far less concern in any surge, and the overall pressure on any hospital system is not the same as before, the importance of still performing surveillance is paramount. While the inflowing data are not as rich as in 2020, they still provide a better syndromic surveillance data set than for other “similar” diseases, such as Flu, especially if combined with other hospital attributes such as associated hospitalization and positivity. These tools can provide the first indication of any future threat, for example providing the first spatial signature of a new variant. Even more importantly, these same tools can be applied to other diseases and health conditions. Already opioid overdose outbreaks are being investigated in a similar manner. However, arguably the most important justification is for any future emergent pandemic threat. While researchers often discuss the need for pandemic preparedness [38], here we have detailed a living, working model, currently supporting hospital and public health operations, and which can be switched to any new emerging threat within hours. The architecture would remain the same, just the data flow would change. Even the many associated geographic attributes, such as congregate buildings, critical infrastructure and social vulnerability, would serve the same role. By continually monitoring the current (and future) situations of C19, new techniques and data manipulations can also be tried.. In effect, the current pandemic has provided us with an evolving template for any future threat. The challenge is to not let complacency dull that edge.

Limitations. Despite the life-saving outcomes of this work in practice, it is worth addressing some of the potential criticisms which should be a dimension for future consideration. Firstly, GeoMEDD does not directly take into account the underlying population. While this might be problematic for mapping of clusters for only one time-period which might be susceptible to the underlying population density, the counter argument is that the daily interpretation of clusters negates this effect by showing how these clusters evolve and move. In effect we are normalizing by time. From an operational perspective, and for the underpinning of syndromic surveillance, it is again important to stress that seeing where those first emerging cases are located is vital.

The second criticism is how such granular investigation and sharing of results might lead to problems of confidentiality [39,40,41]. There are many aspects to this, and a more detailed paper considering this type of response is required. However, we have taken every step in the presentation of results, at meetings and in this paper, to not reveal sensitive information. We have also limited town and neighborhood identification so as not to stigmatize areas. However, at some point, effective operational action requires detailed on the ground information. Where this is not a problem for hospitals and health departments who are custodians of these data, and their responding teams are internal, it has other implications for community operations and FQHCs that in many ways are the front line against the disease. While there is understandable concern about the correct interpretation of maps and data, it is these groups that best know the local situation and the local influencers that could help incrementally with response. The potential stigmatizing of cohorts or neighborhoods should also be considered. At the beginning of the pandemic, there was concern about unwarranted media interest, e.g., around care homes having positive cases. The question is: do we still stigmatize those buildings in the same way, or has the situation been normalized enough that society now wants accurate information? For example, a recent large care home outbreak identified by the team was discussed on the care home’s own social media page in order to warn relatives to stay away. With such homogenous spread, arguably, the biggest problems are now political and not epidemiological in nature. Again, this is an interesting (and geographic) question that needs to be more fully researched.

## 4. Conclusions

The work presented here is the result of a scientific-operational collaboration and therefore novel in its contribution as it expands beyond science alone. The development, application, and lessons learned from GeoMEDD present proof of principle and the higher level thinking needed during a collaborative and geographic response to C19. This should be seen as a template that can be replicated for other hospitals and health systems. The specific contribution this paper makes is that the skills, the conceptual thinking, and arguably the “training” from working in such a traditional research environment has allowed for new spatial operation approaches to be developed. However, the successful use of such advances relies on so much more than the technology alone. There are certainly philosophical considerations as well. For example, the structure of health systems as competitive and fragmented as in the U.S. model, versus cooperative and integrated as in most other developed countries, will impact the ability to have a complete map to inform operations for any emergent disease. Beyond the completeness and timeliness of the data is also the question of how to perform meaningful analysis. Most spatial analytical approaches have been developed solely within the realm of science for scientific application and therefore rely on tests of significance to identify a pattern. C19 has raised awareness that when science meets operational needs in health care, statistical significance may be less relevant than ethical significance (e.g., if you can see one positive case of disease in a building with vulnerable populations, waiting to achieve statistical significance will result in an ethical and moral failure of humanity as sickness or death could have been prevented). Finally, from this philosophical and ethical lens, how we can take what we have learned from such a geographically and temporally granular disease surveillance system and apply it to other diseases, both infectious and chronic. We are having these discussions now and have already demonstrated potential for gynecological cancer [42]. Moving to a practical perspective, our experience suggests that any hospital or health system can benefit from integrating mapping of relevant dimensions of their data to improve operational efficiency and ultimately improve care. While this paper provides conceptualization, methods, and examples, the technology is also posted so that it is freely available to use. It is not hyperbole to state that these advances have helped change the course of the pandemic in the region, saving lives in the process, as a combined academic, hospital, health and community team sought to reduce the local impact of the pandemic rather than just reporting on it. This could only have been achieved by synthesizing the knowledge of our group which included spatial analytics, map communication, database design and programming, health analytics and emergency care while also appreciating the benefit of mixed methods and contextual importance. These skills are always available in typical hospital and health department data analytics teams. What we have shown here is the benefit of building such teams through the collaboration with spatial scientists willing to become more involved in real time analyses. In so doing we build society’s capacity to respond to future pandemics.

## Data Availability

Not applicable.
